# Research ethics in practice: An analysis of ethical issues encountered in qualitative health research with mental health service users and relatives

**DOI:** 10.1007/s11019-023-10169-5

**Published:** 2023-08-28

**Authors:** Sarah Potthoff, Christin Hempeler, Jakov Gather, Astrid Gieselmann, Jochen Vollmann, Matthé Scholten

**Affiliations:** 1 Institute for Medical Ethics and History of Medicine, Ruhr University Bochum, Markstr. 258a, 44799 Bochum, Germany; 2 Department of Psychiatry, Psychotherapy and Preventive Medicine, LWL University Hospital, Ruhr University Bochum, Bochum, Germany; 3 Department of Psychiatry and Psychotherapy, Campus Benjamin Franklin, Charité, Berlin, Germany

**Keywords:** Research ethics, Qualitative research, Institutional review board, Vulnerability, Informed consent, Mental capacity, Voluntariness, Data protection, Mental healthcare, Psychiatry

## Abstract

The ethics review of qualitative health research poses various challenges that are due to a mismatch between the current practice of ethics review and the nature of qualitative methodology. The process of obtaining ethics approval for a study by a research ethics committee before the start of a research study has been described as “procedural ethics” and the identification and handling of ethical issues by researchers during the research process as “ethics in practice.” While some authors dispute and other authors defend the use of procedural ethics in relation to qualitative health research, there is general agreement that it needs to be supplemented with ethics in practice. This article aims to provide an illustration of research ethics in practice by reflecting on the ways in which we identified and addressed ethical and methodological issues that arose in the context of an interview study with mental health service users and relatives. We describe the challenges we faced and the solutions we found in relation to the potential vulnerability of research participants, the voluntariness of consent, the increase of participant access and the heterogeneity of the sample, the protection of privacy and internal confidentiality, and the consideration of personal and contextual factors.

## Introduction

Qualitative health research involves the investigation of personal health, health services, and public health by means of the methods of the social sciences (Green and Thorogood 2014). According to article 23 of the Declaration of Helsinki, all research protocols involving human subjects must be submitted to an independent research ethics committee for approval before the study begins (World Medical Association 2013). Many authors have criticized the current practice of ethics review of qualitative research by research ethics committees (Allbutt and Masters 2010; Israel 2015; Øye et al. 2016; Pollock 2012; Stevenson et al. 2015; Thompson and Harper 2012; Thompson and Russo 2012; Van den Hoonaard 2011; Van den Hoonaard and Hamilton 2016). Authors observe that the ethics review procedures of research ethics committees tend to be tailored to quantitative biomedical research, resulting in a mismatch between ethics review criteria and qualitative research methods (Bell et al. 2014; Dingwall 2008; Israel 2015; Van den Hoonaard 2011; Van den Hoonaard and Hamilton 2016). Because of this mismatch, many authors consider current ethics review procedures as too inflexible and static to be able to guide qualitative health research (Dingwall 2008; Gillam et al. 2009; Guillemin and Gillam 2004; Pollock 2012; Stevenson et al. 2015).

The mismatch cannot be fixed easily because it is grounded in core features of the ethics review process and qualitative methodology. Since research ethics committees must review a research study before it begins, they need a fixed research protocol as a basis for the ethics review, and the Declaration of Helsinki furthermore states that “no amendment to the protocol may be made without consideration and approval by the committee” (World Medical Association 2013). These requirements are difficult to reconcile with the essentially dynamic nature of qualitative research.

The method of theoretical sampling is a case in point. According to this method, the sampling strategy of a study should depend on the results of the analysis of the first data collected (Charmaz 2006; Strauss 1987). This implies that the inclusion criteria and the sample size of studies using theoretical sampling cannot be determined conclusively before the study begins. In many qualitative research designs, furthermore, research questions and study interventions (e.g., the interview guide) can and should change gradually during data collection and analysis (Charmaz 2006; Creswell and Poth 2018; Denzin and Lincoln 2018; Flick 2010; Strauss 1987). While some research ethics committees have developed flexible procedures to accommodate for repeated amendments to research materials, full implementation of such procedures is the exception rather than the rule. In most contexts, then, submitting multiple formal amendments to the research ethics committee for repeated changes to the research protocol seems hardly feasible.

In view of the mismatch between the principles of ethics review and the nature of qualitative methodology, some authors argue that the current practice of ethics review poses serious barriers to scientifically and socially important qualitative health research while offering no genuine protection to research participants in return (Allbutt and Masters 2010; Dingwall 2008; Haggerty 2004; McCormack et al. 2012; Øye et al. 2007; Van den Hoonaard 2011; Van den Hoonaard and Hamilton 2016). This criticism implicitly presupposes a paradigm of research oversight that Emanuel and Grady (2007) call “regulatory protectionism”. Regulatory protectionism implies a focus on the protection of research participants and a rejection of the idea that researchers can oversee their own research and safeguard the rights and interests of their research participants. It assigns responsibility for research oversight to independent research ethics committees and formalizes the informed consent process and the ethics review process. The paradigm of regulatory protectionism can be considered paternalistic in virtue of its tendency to exclude so-called “vulnerable groups” from research participation (Emanuel and Grady 2007).

Responding to the criticism of the ethics review of qualitative health research, other authors contend that the mismatch is overstated and argue for a more positive role for research ethics committees in regulating and overseeing qualitative health research (Carniel et al. 2022; Jennings 2012; Wassenaar and Mamotte 2012), pointing to alternative paradigms of research oversight like those referred to by Emanuel and Grady (2007) as “participant access” and “collaborative partnership.” These paradigms both aim to prevent the exclusion of underrepresented individuals from research but do so in different ways: whereas the former strives to secure research access for vulnerable individuals by reference to rights to autonomy, the latter goes further by involving members of the research participant community in a collaborative research process (Emanuel and Grady 2007).

Both sides of the debate agree, however, that oversight by an independent research ethics committee is not sufficient to ensure that qualitative health research is carried out in ethically justifiable ways. Independent oversight must be complemented with sensitivity among qualitative health researchers to ethical issues arising during the research process and ethical skills in adequately addressing these issues. Examples of models that provide guidance to researchers in this respect are Guillemin and Gillam’s (2004) notion of researchers’ reflexivity and Cascio and Racine’s (2018) person-oriented research ethics approach.

Guillemin and Gillam (2004) describe the process of obtaining ethics approval for a study by a research ethics committee before the study begins as “procedural ethics” and the identification and handling of ethical issues by researchers during the research process as “ethics in practice.” Whereas some might consider procedural ethics and ethics in practice as dichotomous or even mutually exclusive, Guillemin and Gillam (2004) as well as Cascio and Racine (2018) emphasize that their ethics in practice approaches should complement rather than replace the regulatory approach. In a similar vein, Jennings (2012) claims that “the ethics review of social science research is best conceived of as a tool for improving ethical practice in research” (88), and then explains, “it is best seen as a way of ensuring that ethical issues have been considered, and as such the most important part of the review is what happens before the application is submitted” (91). Models for research oversight that aim to accommodate for flexible research designs and the inclusion of underrepresented individuals have been proposed in the literature (Friesen et al. 2017, 2022; Emanuel and Grady 2007).

This article aims to provide an illustration of research ethics in practice by reflecting on the ways in which we identified and addressed ethical and methodological issues that arose in the context of an interview study with mental health service users and relatives. We will first present the details of the interview study that we carried out and then reflect on the ways in which we addressed ethical and methodological issues related to the potential vulnerability of research participants, the voluntariness of consent, the increase of participant access and the heterogeneity of the sample, the protection of privacy and internal confidentiality, and the consideration of personal and contextual factors. An overview of the issues we encountered and the solutions we found is given in Table 1.

**Table 1:** Overview of themes, issues, and solutions

Theme	Issues encountered	Solutions found
Vulnerability of participants	unjustified exclusion of the people concerned from research	including having a serious mental health condition among the inclusion rather than exclusion criteria
	institutionalization	including being under involuntary commitment among the exclusion criteria
	compromised capacity to consent	evaluating the emotional and symptomatic stability of inpatients
		using a capacity screening instrument before obtaining consent
		carrying out a structured capacity assessment if needed
	necessity of special safeguards	determining and specifying the risk-benefit profile of the study
		including detailed information about the risks and benefits of the study in the participant information
		offering the opportunity for professional aftercare
Voluntariness of consent	therapeutic misconception	ensuring that the gatekeeper and interviewer is not the treating physician
		explicitly informing participants that the intervention involves research and not treatment
		clinician explicitly taking the role of researcher during data collection
	dependency and power relations	ensuring that the gatekeeper and interviewer is not the treating physician
		emphasizing the possibility to decline or discontinue participation without experiencing disadvantages
Participant access and sample heterogeneity	potential predominance of positive attitudes toward the mental health system	expanding the sampling strategy to prospective research participants outside the mental health system
	homogeneity due to snowball sampling	assessing participants’ attitudes toward the mental healthcare system following a theoretical sampling strategy
	predominance of self-confident voices	establishing first contact based on both participants’ and researchers’ initiative
Privacy and confidentiality	possibility of general data breaches	developing a data management plan
	demands for data retraction	carrying out pseudonymization of the data and removing identifiable information
		informing participants of the opportunity to have their data retracted until publication of the findings
		giving the contact information of the PI to communicate demands for data retraction
	internal confidentiality	excluding dyads (not our preferred solution)
		informing dyads of risks related to internal confidentiality and obtaining their consent
		analyze dyadic findings at a group level rather than an individual level
Contextual and personal factors	choice of interview setting	considering logistical practicality for participants
		honoring participants’ preferences regarding the interview setting
		providing emotional support in sensitive settings
		offering the opportunity for professional aftercare

## The case study

The current reflection is based on discussions that we had among team members while designing and carrying out an interview study with mental health service users and relatives following a grounded theory approach according to Corbin and Strauss (2015). The study was carried out in Germany between 2019 and 2020 and was part of SALUS, a large interdisciplinary research project on coercion in mental healthcare. The research team was interdisciplinary and included researchers with backgrounds in medical ethics, medicine, philosophy, psychiatry, and sociology. All researchers had clinical experience: one as a psychiatrist, one as a resident in psychiatry, two as auxiliary nurses, and one as an intern.

The aim of the study was to explore service users’ and relatives’ experiences of psychological pressure in mental healthcare services. Psychological pressure encompasses communicative strategies used by professionals and relatives to influence the decision-making of service users and improve their adherence to recommended treatment or social rules (Potthoff et al. 2022). Notable strategies are persuasion, interpersonal leverage, inducements, and threats (Szmukler and Appelbaum 2008). While all these types of treatment pressure involve proposing that a service user do *x* (e.g., give consent to treatment), they influence the service user’s decision-making around accepting the proposal by different means: persuasion entails providing rational arguments in favor of accepting the proposal; interpersonal leverage involves communicating that one will change one’s emotional attitudes toward the person if they refuse the proposal; inducements imply signaling that one will make the person better off if they accept the proposal; and threats involve announcing that one will make the person worse off if they refuse the proposal (Potthoff et al. 2022; Szmukler and Appelbaum 2008).

We collected the data by means of semi-structured interviews with 14 mental health service users and 11 relatives of service users. Interviews lasted between 45 and 120 minutes. Service users who participated in the study had a self-reported psychiatric diagnosis and experience with formal coercion in mental healthcare. Formal coercion encompasses involuntary hospital admission, involuntary treatment, and coercive measures like isolation, mechanical restraint, and physical restraint. Service users who satisfied the abovementioned criteria but were under involuntary commitment at the time at which we conducted interviews were not included in the study. Relatives were informal caregivers of service users who satisfied the abovementioned criteria but were themselves not enrolled in the study. Study participants varied in terms of age, gender, self-reported psychiatric diagnosis, and experience with coercion.

All participants gave written informed consent to participation in the study after having been informed about the details of the study and all other relevant information, both orally and in writing. The study received ethics approval from the research ethics committee of the medical faculty of the Ruhr University Bochum, registration number 18-6584-BR.

## Assessing vulnerability

A key issue in the debate on the ethics of qualitative health research is the tension between the inclusion and the protection of “vulnerable” individuals (Allbutt and Masters 2010; Bell et al. 2014; Bracken-Roche et al. 2016; Holland 2007; Peter and Friedland 2017). Article 19 of the Declaration of Helsinki states that vulnerable groups and individuals “may have an increased likelihood of being wronged or of incurring additional harm” and therefore “should receive specifically considered protection” (World Medical Association 2013). The guidelines of the Council for International Organizations of Medical Sciences (CIOMS) mention the following special safeguards as examples: including vulnerable individuals only in studies that carry no more than minimal risks, supplementing the vulnerable individual’s consent with that of their legal representative, and the application of other tailored safeguards to minimize risk (CIOMS, 2016).

Although the CIOMS guidelines recognize that “vulnerability involves […] also aspects of the ongoing participation in research studies” (2016: 57), research ethics committees commonly require that the vulnerability of prospective research participants be defined and determined before the study begins (Allbutt and Masters 2010; Haggerty 2004; Holland 2007; Peter and Friedland 2017). Since individual research participants are unknown at this stage, research ethics committees are likely to assess the vulnerability of prospective research participants based on group characteristics (Bell et al. 2014).

Our research protocol included prospective research participants with mental disorders, and research ethics committees often assume that people with mental disorders as a group are intrinsically vulnerable (Bell et al. 2014). Since our research protocol specified experience with formal coercion in mental healthcare as an additional inclusion criterion, prospective research participants were likely to have a serious mental illness. We anticipated that this would make it even more likely for the research ethics committee to consider prospective participants of our study as vulnerable.

We were concerned that such an assessment could lead to the unfair exclusion of people with serious mental illness from research on a practice that deeply concerns them. Several authors have pointed out that identifying vulnerability with group characteristics may entail additional stigmatization of people who are already stigmatized (Bell et al. 2014; Bracken-Roche et al. 2016; Carlsson et al. 2017; Øye et al. 2016; Scholten et al. 2021; Schrems 2014). Reconsidering attributions of vulnerability to prospective research participants with mental disorders can thus be conducive to reducing biases and stereotypes. For these and other reasons, the CIOMS guidelines aim to “avoid considering members of entire classes of individuals as vulnerable” and focus on “the specific characteristics that may render individuals vulnerable” (2016: 57). Two of the specific characteristics mentioned in the CIOMS guidelines were relevant to our study: limited capacity to give or withhold consent to research participation and being institutionalized in a mental health hospital.

We considered institutionalization first. The CIOMS guidelines clarify that being confined to a mental health hospital may compromise the voluntariness of consent. Because people who have been involuntarily committed lack alternatives and are strongly dependent on the treatment team, they may feel pressured to consent to research participation. Having discussed this risk, we decided to add being involuntarily committed to a mental health hospital as an exclusion criterion for our study. We considered this exclusion criterion justifiable because the inclusion of people with mental disorders who are involuntarily committed to a mental health hospital was not necessary to achieve the aims of our study. These could also be achieved by interviewing people with experience of coercion who are currently in a community or voluntary inpatient setting.

We subsequently considered limited capacity to give consent. Many discussions on vulnerability revolve around the capacity of people with mental disorders to give informed consent (Bell et al. 2014; Bracken-Roche et al. 2016; Peter and Friedland 2017). We were aware that a lack of capacity to give consent cannot be inferred from a person’s psychiatric diagnosis (Grisso and Appelbaum 1998; Kim 2010; Scholten et al. 2021). At the same time, we recognized that conditions like psychosis and mania are risk factors for impaired capacity to give consent (Kim 2010: 40–54).

One strategy we considered to address this was carrying out a structured capacity assessment when obtaining informed consent from prospective research participants. Given the so-called presumption of capacity (Grisso and Appelbaum 1998; Kim 2010) and the relatively low risks attached to participation in our interview study, we decided against this option and resolved to carry out a structured capacity assessment only if prospective research participants displayed risk factors for impaired capacity, such as signs of acute psychosis or mania. We proceeded as follows. A research team member who worked as a consultant psychiatrist evaluated the emotional and symptomatic stability of prospective research participants who were inpatients before they were approached for research participation, and only individuals who were assessed as sufficiently stable were approached for research participation. During the informed consent process, this research team member assessed whether prospective research participants displayed risk factors for impaired capacity. We judged that a similar psychiatric screening was not necessary for service users who were interviewed in a community setting because these service users can be assumed to be sufficiently stable, and because the researchers who carried out the interviews had sufficient knowledge about risk factors for impaired capacity to consent.

Since none of the prospective research participants displayed risk factors for impaired capacity, we did not carry out structured capacity assessments during the research process. We did give special consideration to explaining the details of the study in a way that was understandable to individual research participants and ensuring that this information was understood. We prepared the information disclosure carefully and paid special attention to the needs of prospective research participants during the informed consent process. Bracken-Roche et al. (2016: 337) note that “relational sources of vulnerability” include not only “participants’ education level and health literacy” but also “suboptimal study designs, researcher biases, and a lack of support”. Acknowledging this, we prepared the interview carefully and stayed attuned to the cognitive and emotional needs of research participants during the interview.

Finally, we considered which other safeguards might be necessary. We recognized that the desirability of special safeguards for vulnerable research participants is subject to debate (Allbutt and Masters 2010; Bracken-Roche et al. 2016; Carlsson et al. 2017; Cox and McDonald 2013; Gieselmann et al. 2019; Øye et al. 2016; Scholten and Vollmann 2019; Schrems 2014; Smith 2008; Thompson and Chambers 2012). Acknowledging that special safeguards are needed to minimize risks to vulnerable research participants, authors have argued that a focus on risks can become problematic when benefits to participants are underestimated or simply overlooked (Allbutt and Masters 2010; Carlsson et al. 2017; Cox and McDonald 2013; Graham et al. 2007; Holland 2007; Kars et al. 2016; Lewis and Graham 2007; Witham et al. 2015). Benefits to research participants should be considered and weighed against the risks.

When discussing our research protocol, we identified the following risks to individual research participants: experience of emotional distress due to being confronted with sensitive topics or having to face traumatic experiences, breach of privacy, and negative consequences of a breach of privacy (e.g., impaired relationships with clinicians, relatives, or other parties). Likewise, we identified the following potential benefits to individual research participants: having the opportunity to give one’s opinion about the use of coercion in mental healthcare, having the opportunity to reflect on experiences with coercion in mental healthcare, contributing to the generation of scientific knowledge, and indirectly contributing to the improvement of mental healthcare services and quality of care. We also determined that our study had no therapeutic benefits to individual research participants. Although study participation could potentially be conducive to recovery, this effect was neither expected nor aimed for.

After having determined the risk-benefit profile of our research study, we included the experience of substantial emotional distress among the discontinuation criteria of the interview. As an additional safeguard, we offered research participants the opportunity to talk with the research member who was a consultant psychiatrist after the interview to process possible emotional distress. Finally, we included the risk-benefit profile in the ethics application and in the disclosure information for prospective research participants.

## Ensuring voluntariness

In qualitative research, researchers must be sufficiently close to research participants to be able to generate rich data, yet at the same time keep a suitable distance to prevent undue influence and respect privacy (Guillemin and Gillam 2006; Guillemin and Heggen 2009; Peter and Friedland 2017). Special considerations apply in qualitative health research, where researchers regularly assume the dual role of researcher and health professional. This dual role amplifies the tension between closeness and distance (Carlsson et al. 2017; Graor and Knapik 2013; Keogh and Daly 2009; Lawrence et al. 2012; Thompson and Harper 2012; Thompson and Russo 2012).

One issue that we discussed in this regard was whether it was ethically desirable to look for research participants in the mental healthcare institution where one of the research team members worked as a psychiatrist. An advantage of this recruitment method was that the research team member would be acquainted with prospective research participants and hence in the position to assess whether they satisfied the inclusion criteria and whether they displayed risk factors for impaired capacity to give consent. At the same time, this method gave rise to both ethical and methodological issues.

One ethical issue that we discussed in relation to the voluntariness of consent was the possibility that prospective research participants mistake the interview for a therapeutic session with potential positive effects on their mental health and hence agree to participate in the study for the wrong reason. This conflation of research and therapy is commonly referred to as the “therapeutic misconception” (Appelbaum et al. 1982). We minimized the risk of this misconception in three ways. First, we ensured that the research team member did not serve as the treating psychiatrist of prospective research participants. Second, we informed prospective research participants explicitly that the interview was a study intervention and not a therapeutic conversation. Third, when carrying out the interviews, the research team member explicitly maintained the role of researcher (Brinkmann and Kvale 2005; Taquette and Da Borges Matta Souza, 2022).

Another ethical issue that we discussed was that prospective research participants might feel pressured to participate in the study due to possible relationships of dependency with the research team member. Here, too, we found it important that the researcher did not serve as the treating physician of prospective research participants. Furthermore, we made it explicit during the informed consent process that prospective research participants could decline or discontinue study participation without reprisal of any sort. In this way, we found seven service users who were prepared to participate in the study. There were also service users who declined research participation, which can be interpreted as a sign that service users did not feel under pressure to participate.

## Increasing participant access and sample heterogeneity

The chosen sampling strategy of approaching inpatients via the clinician-researcher also raised a methodological concern. We discussed the possibility that this sampling strategy would result in an insufficiently heterogeneous sample. The research team member who worked as a psychiatrist at the mental health hospital in effect served as a gatekeeper for the research study. A gatekeeper has the power to decide who should and should not be included in research and always makes a preselection of potential research participants (Allbutt and Masters 2010; Carlsson et al. 2017; Kristensen and Ravn 2015; Rugkåsa and Canvin 2011). We were concerned that this might lead to a gatekeeper bias: individuals who had a positive relationship with the gatekeeper and a positive attitude toward the mental healthcare system would be more willing to participate in the study than individuals who are critical of the gatekeeper or the services. This could result in a relatively homogeneous sample.

We judged this to be problematic for both methodological and ethical reasons. The aim of our study was to evaluate the use of informal coercion and psychological pressure in mental healthcare from a service user perspective, and it is well-known that service users’ attitudes toward coercion vary greatly. To avoid a gatekeeper bias in the selection of research participants and ensure the heterogeneity of the sample, we thought it necessary to enroll service users with negative experiences with or a critical attitude toward mental healthcare services.

We proceeded as follows. We expanded our sampling strategy and included service users who were not previously known to our research group. We contacted self-help groups in various cities and asked them if they could forward the information leaflet of our study to their members. In this way, we found seven additional research participants. We had no prior contact with these participants, and they were not in inpatient treatment at the time of the interview. They contacted us via email on their own initiative. Some of them knew each other and shared the information about our research study amongst each other. This led to the use of snowball sampling.

Since snowball sampling is based on self-selection and peer recommendation, the second part of the sample could also turn out to have a relatively high level of internal homogeneity. To avoid this and guarantee sufficient heterogeneity, we explored research participants’ attitudes toward the mental healthcare system during data collection and data analysis. Self-selection furthermore implies a high level of initiative on the part of research participants. It is likely that service users who contacted us must have viewed their own experience and knowledge as relevant and important enough to share it in the scientific community. One consequence of this is that people with less confidence in the relevance and importance of their narratives may not be represented in the sample (Kristensen and Ravn 2015; Rugkåsa and Canvin 2011). The complementary sampling method via the gatekeeper provided a counterweight to this. By approaching service users personally in the inpatient setting, we were able to include service users who might not have contacted us of their own accord.

## Securing privacy and internal confidentiality

We developed a data management plan to secure the privacy of research participants. Although the plan was relatively standard for qualitative research, it is nonetheless helpful to briefly summarize its key points. We audiotaped the interviews, transcribed the interview material ad verbatim, and replaced any identifiable interview material (e.g., names, places, institutions, and special biographical data) with placeholders before the start of the data analysis. We pseudonymized the data by assigning a random code to both the consent form containing identifiable information and the respective transcript and record of demographical data. We stored the consent forms securely and separately from the other study data according to the European General Data Protection Regulation. Upon publication of the findings, we destroyed all personal and identifiable information, and stored the transcripts securely for a period of at least ten years.

The pseudonymization of the data allowed us to retrieve and delete data should participants withdraw their consent to study participation before publication of the findings. We mentioned this option explicitly in the information leaflet and included the name and contact details of the principal investigator to ensure that participants had a real opportunity to withdraw their consent even after the interview. One research participant contacted the principal investigator a couple of days after the interview because they felt they had made too personal statements about family members. The principal investigator pointed the research participant to the option of withdrawing consent, and they decided to make use of this option. We thereupon retrieved and deleted all data belonging to the research participant in accordance with the data management plan.

We also came across more complicated privacy issues during the research process. According to grounded theory methodology, we aimed to reconstruct various perspectives on psychological pressure in mental healthcare to attain a comprehensive understanding of the phenomenon. Based on anecdotal evidence and the literature, there was reason to assume that service users experience psychological pressure not only in mental health hospitals but also in their personal social environment. For this reason, we wanted to explore the perspectives of both service users and close relatives. We thus developed the idea to include dyads in our study.

After we submitted our application for ethics approval, the research ethics committee expressed reservations about the inclusion of dyads in the study. The explanation was that conducting interviews with dyads would make it difficult to maintain confidentiality and avoid harm to research participants. The type of confidentiality under discussion was internal confidentiality. Internal confidentiality differs from external confidentiality in that it “refers to the possibility that research participants involved in a common study will be able to identify one another on the basis of published information” (Ummel and Achille 2016: 807). We shared the research ethics committee’s concern that interviews with dyads could potentially breach internal confidentiality and damage relationships between study participants. Having discussed the report from the research ethics committee, we decided to refrain from including dyads in the study, amended the study protocol, and submitted a revised ethics application, which was then approved.

Looking back, however, we are unsure whether concerns about internal confidentiality should have been decisive. A look at a situation we encountered during our study might help to explain why. After we distributed information about our study, a relative of a service user contacted us and expressed the wish to participate in the study. She had a daughter who had a mental disorder and previous experience with coercion during inpatient stays. After we scheduled an appointment for the interview, the mother informed us that her daughter would like to take part in the study as well and that she herself supports this. Given our amended study protocol, however, we had to tell the mother and the daughter that only one of them could participate in the study. The prospective research participants then had to decide who would participate and who would be excluded. They decided that the mother would participate. The research ethics committee’s prior and hence necessarily non-contextual demand seemed to impair rather than protect the relationship between the mother and daughter. The precautionary requirement that dyads not be included thus seemed problematic in this case.

The source of the problem is that the research ethics committee had to assess the likelihood of a breach of internal confidentiality before the study began, without having full information about the research participants and about how the analyzed data would be prepared for publication. The committee’s precautionary approach is understandable in view of this. One question that could be raised, however, is whether dyads should not have been given the opportunity to decide for themselves whether they find the risk that their relative can trace information in the publication back to them acceptable, or whether they would mind this at all. Relevant in this respect is that research participants can still withdraw from the study after the interview, as described above. Moreover, there are methods of data analysis that allow researchers to report findings from dyads while maintaining internal confidentiality. One method is to analyze and report the dyadic findings at a group level rather than at the level of individual dyads (Forbat and Henderson 2003; Tolich et al. 2020; Ummel and Achille 2016).

In our study, the issue of maintaining internal confidentiality went beyond the question of whether dyads could be included. Given our partial snowball sampling method, a substantial share of the service users who contacted us based on the information we distributed among self-help groups knew each other and recommended participation amongst each other. We ensured internal confidentiality by keeping the analysis of the data and the report of the findings on a conceptual-theoretical level in accordance with grounded theory methodology. We believe that this would have been possible for the dyadic findings as well.

## Considering personal and contextual factors

Another issue we discussed during team meetings was the necessity to consider personal and contextual factors in choosing the interview setting. The setting of an interview is important because it influences the nature and quality of the data that is collected (Gubrium et al. 2012; Holstein and Gubrium 1995, 2016; Miller 2016). While from a methodological perspective qualitative researchers must cultivate a certain level of closeness to research participants to be able to generate rich data, from an ethical perspective they must keep a suitable distance to prevent undue influence and respect the privacy and integrity of research participants (Guillemin and Gillam 2006; Guillemin and Heggen 2009; Kvale and Brinkmann 2009; Peter and Friedland 2017).

The settings we discussed were a room at the mental health hospital, a room at the university, and the homes of research participants. Researchers in our interdisciplinary research group assumed different default options based on what is customary in their discipline. While in medicine it is common practice to carry out research interviews at the hospital, in social science they are usually carried out in settings such as participants’ homes, workplaces or public places. Team members with a background in the humanities thought it most natural to conduct the interviews at the university.

Team members with a background in social science emphasized that carrying out interviews in participant-selected settings reduces the potential power imbalance and forges a relationship of trust between researchers and participants (Brinkmann and Kvale 2005; Elwood and Martin 2000; Pascoe Leahy 2022). They furthermore pointed out that it enables a contextualization of the data through the researcher’s insight into parts of participants’ everyday life (Charmaz 2006; Gubrium et al. 2012; Holstein and Gubrium 1995, 2016). It was precisely this deeper insight into participants’ everyday life that team members with a background in medicine found potentially intrusive.

We opted for a hybrid solution. For the interviews with participants who were inpatients, we reserved a room in the research department of the mental health hospital. An advantage of this was that participants did not have to commute for the interview. The prospect of an interview at home or on the university campus would put an additional demand on prospective participants and thus raise the threshold for research participation. By contrast, we asked the research participants who contacted us via self-help groups where they would prefer to have the interview.

Of the seven service users who contacted us via the self-help groups, five preferred to have the interview at home, one preferred to have it at the mental health hospital and one at the university. Interviews at home were conducted by two researchers. The participant who expressed a preference for an interview at the mental health hospital later voiced concerns about this location because she had negative experiences with mental health hospitals. We emphasized that she was free to choose another setting for the interview, but after thoughtful consideration she decided that she wanted to have the interview at the mental health hospital to be able to connect more positively to this setting. We decided to honor this wish after consultation within the research team. Although several scholars have pointed out that interview topics that evoke strong affective responses do not necessarily impose harm on research participants (Graor and Knapik 2013; Pascoe Leahy 2022), the interviewer pointed the participant to the possibility to talk to the research team member who is a psychiatrist after the interview to process possible emotional distress. The participant decided to make use of this oppurtunity. Notwithstanding the sensitive topics discussed during the interviews, participants appreciated having the opportunity to voice their experiences and express their opinions on the use of coercion and psychological pressure in mental healthcare.

## Conclusion and recommendations

We have reflected on ethical and methodological issues that arose during a qualitative interview study with mental health service users and relatives, and described the ways in which we addressed these issues in our research practice. While procedural ethics and ethics in practice are often seen as two separate domains between which there is no interaction, our analysis shows that independent ethics review and ethics in practice can usefully complement each other. Having to prepare an application for ethics approval of our research study by the research ethics committee urged us to think through our research process, and this enabled us not only to identify and address ethical issues in advance but also to enhance our competence in research ethics in practice.

Although every research study is unique, we hope that our description of the real-life ethical challenges can usefully complement ethical guidelines for qualitative health research and be of help to qualitative health researchers and members of research ethics committees. Our experiences support a transition from the paradigm of regulatory protectionism to the paradigms of participant access and collaborative partnership (Emanuel and Grady 2007). While the solutions we have described in this article generally remain within the paradigm of participant access, we have complemented it with a commitment to reflexivity (Guillemin and Gillam 2004) and a person-oriented research approach (Cascio and Racine 2018). Based on our experiences during the research study and our more general commitment to participatory research, we decided to move toward collaborative partnership and established a peer advisory board consisting of service users and relatives of service users for our ongoing research project SALUS.

The following more general lessons can be drawn from the analysis of our experiences. Based on our analysis, we would advise research ethics committees to:

- actively cultivate a self-understanding as a contact point for consultation and advice rather than a mere regulatory oversight body;
- consider vulnerability as a functional and dynamic concept relating to individual and contextual factors rather than group characteristics;
- respond to research studies that involve potentially vulnerable individuals by considering suitable safeguards rather than excluding vulnerable groups altogether;
- discuss which safeguards are suitable jointly with researchers;
- develop flexible and swift amendment procedures to accommodate for open and dynamic research designs; and
- offer consultation and advice not only before but also during a research study.

These recommendations can be complemented with recommendations for qualitative health researchers. Based on our analysis, we would advise researchers to:

- consider the ethics review process not as a bureaucratic hurdle but rather as an invitation to think through the full research process in order to anticipate and address potential ethical challenges;
- strive to be as transparent as possible when preparing the ethics application with the aim of enabling the research ethics committee to evaluate potential ethical issues and providing a starting point for discussion;
- cultivate ethical sensitivity within one’s own research team and engage in ethics in practice both before and during the research process; and
- discuss ethical challenges openly with co-researchers and the responsible research ethics committee.
